# The Namaqua rock mouse (*Micaelamys namaquensis*) as a potential reservoir and host of arthropod vectors of diseases of medical and veterinary importance in South Africa

**DOI:** 10.1186/1756-3305-7-366

**Published:** 2014-08-15

**Authors:** Dina M Fagir, Eddie A Ueckermann, Ivan G Horak, Nigel C Bennett, Heike Lutermann

**Affiliations:** Mammal Research Institute, Department of Zoology and Entomology, University of Pretoria, Private Bag X20, Hatfield, 0028 South Africa; ARC-Plant Protection Research Institute, Private Bag X134, Queenswood, Pretoria, 0121 South Africa; School of Biological Sciences/Zoology, North-West University, Potchefstroom, 2520 South Africa; Department of Veterinary Tropical Diseases, Faculty of Veterinary Science, University of Pretoria, Onderstepoort, 0110 South Africa

**Keywords:** *Micaelamys namaquensis*, Flea, Tick, Vector, *Bartonella*, *Rickettsia*, Zoonotic disease

## Abstract

**Background:**

The role of endemic murid rodents as hosts of arthropod vectors of diseases of medical and veterinary significance is well established in the northern hemisphere. In contrast, endemic murids are comparatively understudied as vector hosts in Africa, particularly in South Africa. Considering the great rodent diversity in South Africa, many of which may occur as human commensals, this is unwarranted.

**Methods:**

In the current study we assessed the ectoparasite community of a widespread southern African endemic, the Namaqua rock mouse (*Micaelamys namaquensis*), that is known to carry *Bartonella* spp. and may attain pest status. We aimed to identify possible vectors of medical and/or veterinary importance which this species may harbour and explore the contributions of habitat type, season, host sex and body size on ectoparasite prevalence and abundance.

**Results:**

Small mammal abundance was substantially lower in grasslands compared to rocky outcrops. Although the small mammal community comprised of different species in the two habitats, *M. namaquensis* was the most abundant species in both habitat types. From these 23 ectoparasite species from four taxa (fleas, ticks, mites and lice) were collected. However, only one flea (*Xenopsylla brasiliensis*) and one tick species (*Haemaphysalis elliptica*) have a high zoonotic potential and have been implicated as vectors for *Yersinia pestis* and *Bartonella* spp. and *Rickettsia conorii*, respectively. The disease status of the most commonly collected tick (*Rhipicephalus distinctus*) is currently unknown. Only flea burdens differed markedly between habitat types and increased with body size. With the exception of lice, all parasite taxa exhibited seasonal peaks in abundance during spring and summer.

**Conclusion:**

*M. namaquensis* is the dominant small mammal species irrespective of habitat type. Despite the great ectoparasite diversity harboured by *M. namaquensis*, only a small number of these are known as vectors of diseases of medical and/or veterinary importance but occur at high prevalence and/or abundance. This raises concern regarding the potential of this host as an endemic reservoir for zoonotic diseases. Consequently, additional sampling throughout its distributional range and research addressing the role of *M. namaquensis* as a reservoir for zoonotic diseases in southern Africa is urgently needed.

## Background

Globally, rodents, particularly members of the murid family, are important hosts for ectoparasitic arthropods, many of which are vectors of diseases of veterinary and medical importance [1]. For example, ticks of the genus *Ixodes*, harboured by several common murid rodents in North America and Europe, are important vectors for diseases such as Lyme borreliosis (*Borrelia burgdorferi* s. l., *B. afzelii*, *B. garinii*), human babesiosis (*Theileria microti*) and human granulocytic ehrlichiosis (*Anaplasma phagocytophilum*) [2–4]. In North and East Africa invasive *Rattus* spp. as well as several endemic rodents are hosts to flea species (e.g. *Xenopssylla cheopis*, *X. brasiliensis* and *Dinopsyllus lypusus*) that are vectors for bubonic plague (*Yersinia pestis*) but also *Bartonella* spp. including *B. elizabethae* that causes human endocarditis [5–11]. Many of the murid hosts of these vectors are also the dominant species in small mammal communities and may attain pest status while these vectors show low host specificity resulting in a high zoonotic potential locally [1, 5, 12, 13].

Within a host population parasites tend to exhibit an overdispersed distribution with only a fraction of the host individuals of a given species harbouring the majority of parasites [14, 15]. These patterns are generated by a suite of factors that can be divided into two general categories, i.e. abiotic (e.g. climate) and biotic (e.g. host sex) factors. Above all, seasonal fluctuations in parasite prevalence and abundance are commonly observed and particularly pronounced in ectoparasitic arthropods. This may be linked to the life-cycle and seasonal fluctuations in temperature and humidity, which often determines the duration of the developmental stages in these parasites [16, 17]. In addition, seasonal patterns may be generated by the susceptibility of such ectoparasites to desiccation during periods spend off-host [16, 18, 19]. Such periods may differ substantially between ectoparasite taxa and ixodid ticks can be encountered during the majority of their life-cycle in the environment. In contrast, many flea and mite species live in the buffered environment of their hosts’ nest when not feeding on the host, whereas the majority of lice spend their entire life-cycle on the host [16, 18, 20]. However, changes in thermoregulatory demands and reproductive activity of the host in response to seasonal climate and fluctuations in food availability may also contribute to the seasonal patterns observed in many ectoparasite species [1].

In addition to seasonal patterns, sex-bias in ectoparasite burdens has been observed across a wide range of animal taxa and appears to be mostly male biased [21, 22]. Such differences have alternatively been linked to body size differences and physiological or behavioural mechanisms [21, 23, 24]. Elevated levels of testosterone have been shown to increase male susceptibility to parasite infestation [25]. Alternatively, but not mutually exclusive, behavioural mechanisms such as sex-specific ranging behaviour or social aggregation that affect exposure and/or transmission may cause sex biases in parasite burden [15, 26].

Although endemic murid rodents have received substantial interest as hosts of ectoparasite vectors of diseases in Africa e.g. [5, 6] such studies remain limited for South Africa and often only consider a single parasite taxon [27–30]. This appears unwarranted given the high densities that some murid populations can achieve locally and the presence of known disease vectors such as *X. cheopis* and *X. brasiliensis* [31]. In addition, a number of tick species with immature stages that may exploit rodents are of great economic importance in the livestock industry of this region including various *Rhipicephalus* spp. that may carry *Theileria* ssp., *Babesia* spp. and *Anaplasma* spp., but also *Rickettsia conorii* causing African tick bite fever in humans [3, 13].

In the current study, we investigated the ectoparasite community of the Namaqua rock mouse (*Micaelamys namaquensis*) in a nature reserve in the Gauteng Province, South Africa. The species has a wide distributional range and occurs with few exceptions in eastern Mozambique across Africa south of the 18° latitude [32]. Rock mice are flexible in their habitat requirements but prefer rocky outcrops or hillsides as indicated by their common name. In their preferred habitat the species dominates the small mammal community and it may occur as commensal in rural communities [33]. They are nocturnal with an omnivorous diet [32]. Little is known about their social system and although some authors describe them as communal [32] females appear to occupy exclusive territories while male territories may overlap with several conspecific of either sex [34]. Rock mice breed during the rainy season which coincides with winter in the western coastal areas of South Africa while they breed during the summer in eastern parts of the country [34, 35]. A large number of ectoparasite species have been reported to infest *M. namaquensis* including 34 flea species from four families (Pulicidae, Hystrichopsyllidae, Leptopsyllidae and Chimaeropsyllidae, [36, 37]), three species of lice from two families (Hoplopleuridae and Polyplacidae [38]), 12 mite species from two families (Laelaptidae and Trombiculidae [39]) and 26 tick species from three families (Ixodidae, Argasidae and Nuttalliellidae [28, 30, 40, 41]), indicating their potential role as both a vector and reservoir host. However, for most of these only species accounts are available with little or no information regarding the locality or number of host individuals sampled and only a single study has sampled the same population on more than one occasion [29]. Rock mice have been identified as one of the preferred hosts for the tick *Haemaphysalis elliptica* that transmits *Babesia rossi* to dogs and wild canids but also *Rickettsia conorii* to humans [13, 28, 29, 42]. Although they are not necessarily the preferred hosts, rock mice may also sustain significant numbers of *Rhipicephalus warburtoni* which carries *Anaplasma bovis* and may also cause paralysis in goats [30, 43]. In addition, *M. namaquensis* has been shown to carry a number of *Bartonella* spp. including *B. elizabethae* in several South African provinces at a prevalence of up to 58% [44, 45]. The aim of the current study was (1) to conduct the first comprehensive assessment of the ectoparasite species parasitizing *M. namaquensis* in a single locality and (2) to identify key ectoparasite species that may be vectors of diseases of veterinary and/or medical importance. In addition, we aimed (3) to investigate the contributions of abiotic (i.e. season) and biotic factors (i.e. host sex) on the distribution of ectoparasite taxa among hosts.

## Methods

Animals were sampled at Telperion/Ezemvelo Nature Reserve (25° 41’ S, 28° 56’ E) using 72 live-Sherman traps (H. B. Sherman Traps, Inc., Tallahassee, Florida) per plot on 16 plots (8 rocky outcrops and 8 grasslands). The study site is located in the summer rainfall region of South Africa (October-April). During the study period a total of 6.4 mm precipitation was recorded from June to September 2010 while it was a minimum of 43.8 mm per month during the remainder of the study period and exceeded 160 mm in April, December and January (SA Weather Service). At the same time, the minimum and maximum temperatures recorded were 10.0 ± 0.3°C and 26.2 ± 0.2°C, respectively. The lowest (−5.6°C) and highest temperatures (36.1°C) were measured in June and November, respectively. Sampling took place five times between April 2010 and April 2011 (April/May, July/August, October/November 2010; January/February, April/May 2011) to cover all seasons. In the study area breeding occurs between October and March [35]. During the first trip (April/May 2010) sampling was limited to five rocky outcrops and one grassland plot. In addition, during the last trip mice were exclusively sampled from rocky outcrops (8 plots). Traps were baited with a mixture of peanut butter and oats and set over night in four parallel lines of 18 traps each approximately 10 paces apart. On each plot traps were set for four consecutive nights and checked around dawn. To limit trap related deaths as a result of environmental exposure, traps were closed during the day and bedding was provided in the traps during winter.

Animals were removed from the traps using Ziplock® bags and hand-restrained during examination. The sex of the animal caught was recorded and the entire body of each individual was carefully searched for the presence of ectoparasite by back-combing the fur with tweezers and blowing in the fur. In addition, ear margins, legs and the base of the tail were also checked for the presence of ectoparasites. The processing surface was covered with white sheets and ectoparasites that dropped or jumped off the host were caught by hand or with tweezers. Furthermore, the handling bag was carefully searched for ectoparasites and cleaned between animals to avoid cross-contamination. All ectoparasites encountered were removed using fine tweezers and stored in 70% ethanol for later counting and identification to species level. The body length of all mice captured was measured from the neck to the base of their tail using callipers. They were marked with ear notches and released at their site of capture in the afternoon. For the current study only the first capture of an individual during a capture period was included. For microscopic examination, fleas, lice and mites were cleared and mounted following the techniques described in [31, 37, 46], respectively. Fleas and lice were identified using the morphological key of [37] and [31], respectively. Mites were identified using [46] and ticks were identified to species or species group using descriptions provided by [13, 47].

All mice captured were considered for analyses and the age of individuals was unknown. However, since body size (measured as body length) may be a proxy for age and to confirm previous reports that body size was similar between the sexes [32] we carried out a generalized linear mixed model (GLMM) including habitat, season and sex as well as the interaction between season and sex as independent variables. Study plot was included as a random effect to account for possible site effects. Since body length was not normally distributed and transformations were unsuccessful (Kruskal-Wallis test: p ≤ 0.05), we fitted a model with a Gamma distribution and log-link function. The results showed that body length varied significantly with season (F4_,197_ = 3.656, p = 0.007), with animals being significantly larger in spring (81.0 ± 2.4 mm) compared to autumn 2010 (73.8 ± 1.9 mm, t = 3.725, p < 0.0001) and autumn 2011 (76.11 ± 2.6 mm, t = 2.015, p = 0.045). There were no significant differences between any other seasons (p ≥ 0.057). This is likely a result of the recruitment of juveniles in the study population at the end of the breeding season. Neither habitat, sex (males: 76.91 ± 1.40, females: 76.45 ± 1.47) nor the interaction between season and sex was significant (p ≥ 0.137).

The prevalence and abundance (as defined by [48]) were calculated for each of the four higher taxa (i.e. fleas, lice, mites and ticks, see results) as well as the individual parasite species found. The effect of season (i.e. April/May 2010: autumn 2010, July/August: winter, October/November: spring 2010; January/February: summer, April/May 2011: autumn 2011) and host sex on prevalence and abundance of the different ectoparasite taxa were investigated using GLMMs fitted with a binomial (prevalence) and negative-binomial (abundance) data distribution, respectively. To account for possible effects of habitat type (grassland vs. rocky outcrop) this variable was added as an independent factor. Capture plot was included as random effect. We added body length as covariate in all GLMMs. All statistical analyses were conducted in IBM SPSS version 21 (IBM SPSS Statistics 21.Ink 2013).

## Results

Throughout the study a total of 358 individuals from eleven small mammal species were captured (Table 1). Namaqua rock mice were the dominant species in both habitat types and comprised 59.2% of all animals captured (Table 1). A total of 212 mice of which 120 males (56.6%) and 92 females (43.4%) were captured and examined for ectoparasites (Table 2). From these 6626 ectoparasites from four taxa were collected. Fleas and immature ticks were the most prevalent parasites recovered followed by mites and lice (Table 3). At the same time, mites were the most abundant taxon recovered (Table 3). Lice occurred at a low prevalence and abundance.

**Table 1:** **Summary of small mammals captured during the study period**

Species	Total	Grassland	Rocky outcrop
*Micaelamys namaquensis*	313 (59.2%)	23 (34.3%)	189 (64.7%)
*Elephantulus myurus*	81 (22.6%)	10 (14.9%)	71 (24.3%)
*Tatera leucogaster*	14 (3.9%)	14 (20.9%)	0
*Crocidura* spp.	13 (3.6%)	2 (3.0%)	11 (3.8%)
*Acomys spinosissimus*	10 (2.8%)	0	10 (3.4%)
*Dendromys* spp.	8 (2.2%)	8 (11.9%)	0
*Rhabdomys dilectus*	7 (1.9%)	7 (10.4%)	0
*Aethomys ineptus*	5 (1.4%)	0	5 (1.7%)
*Lemniscomys rosalia*	5 (1.4%)	2 (3.0%)	3 (1.0%)
*Graphiurus* spp.	3 (0.8%)	0	3 (1.0%)
*Mus minutoides*	1 (0.3%)	1 (1.5%)	0
**Total (individuals)**	**359**	**67**	**292**

Displayed are total counts and the respective percentage in brackets.

**Table 2:** **Summary of individual** ***M. namaquensis*** **captured per trip**

Season	Total	Males	Females
**April 2010**	79	42	37
**July 2010**	17	8	9
**October 2010**	47	29	18
**January 2011**	45	25	20
**April 2011**	24	16	8

**Table 3:** **Summary of the parasite groups found on** ***M. namaquensis*** **and their infection parameters**

Taxon	Total	Prevalence (%)	Mean abundance (±SE)
**Fleas**	1072	78.2	4.96 (±0.423)
**Lice**	508	21.3	2.35 (±0.767)
**Mites**	3301	53.7	15.28 (±3.225)
**Ticks**	1744	78.2	8.07 (±1.336)

### Ectoparasite species

A total of five flea species representing five genera were collected (Table 4). Of these, *Xenopsylla brasiliensis* was by far the most prevalent and abundant flea species (Table 4). However, *Chiastopsylla godfreyi* and *Epirimia aganippes* were also quite common though their abundance was low. In contrast, *Dinopsyllus ellobius* and *Demeillonia granti* only occurred at low prevalence and abundance (Table 4).

**Table 4:** **Summary of the ectoparasite species found and their infection parameters in Namaqua rock mice**

Taxon	Species	Larva	Nymph	Male	Female	Total	Prevalence (%)	Mean abundance (±SE)
Fleas	*Xenopsylla brasiliensis*	-	-	351	240	591	61.2	2.74 (±0.299)
	*Chiastopsylla godfreyi*	-	-	75	113	188	28.7	0.87 (±0.150)
	*Epirimia aganippes*	-	-	91	127	218	26.9	1.01 (±0.171)
	*Dinopsyllus ellobius*	-	-	18	-	18	6	0.08 (±0.024)
	*Demeillonia granti*	*-*	*-*	1	-	1	0.5	0.00 (±0.005)
Lice	*Hoplopleura aethomydis*	*-*	*-*	10	101	111	12	0.51 (±0.20)
	*Hoplopleura patersoni*	*-*	*-*	162	31	193	16.2	0.89 (±0.270)
	*Polyplax praomydis*	*-*	*-*	14	7	21	5.1	0.10 (±0.039)
Mites	Trombiculidae (chiggers)	3001	-	-	-	3001	25.9	13.89 (±3.235)
	*Androlaelaps rhabdomysi*	-	84	33	63	180	20.4	0.83 (±0.231)
	*Androlaelaps marshalli*	-	1	-	-	1	0.5	0.00 (±(0.005)
	*Androlaelaps zuluensis*	-	-	-	32	32	0.5	0.15 (±0.148)
	*Laelaps roubaudi*	-	-	4	27	31	3.7	0.14 (±0.087)
	*Laelaps simillimus*	-	-	-	2	2	0.5	0.01 (±0.009)
	*Laelaps* sp*.*	-	5	11	30	46	5.6	0.21 (±0.136)
Ticks	*R. appendiculatus*	1	1	*-*	*-*	2	0.9	0.01 (±0.007)
	*R. warburtoni/arnoldi*	16	3	*-*	*-*	19	5.1	0.09 (±0.029)
	*R. decoloratus*	3		*-*	*-*	3	1.4	0.01 (±0.008)
	*R. distinctus*	956	306	*-*	*-*	1262	67.1	5.84 (±1.056)
	*R. evertsi evertsi*	4		*-*	*-*	4	1.9	0.02 (±0.009)
	*Rhipicephalus* spp.	2		*-*	*-*	2	0.9	0.01 (±0.007)
	*Haemaphysalis* spp.	382	67	*-*	*-*	449	35.6	2.07 (±0.636)
	*Ixodes spp.*	4	-	*-*	*-*	4	1.4	0.02 (±0.015)

A total of three louse species were recovered of which *Hoplopleura patersoni* was the most prevalent and abundant (Table 4). However, the prevalence of *H. aethomydis* was not much lower while both prevalence and abundance of *Polyplax praomydis* was substantially lower than that of both *Hoplopleura* spp. (Table 4).

Mites were the second most speciose ectoparasite taxon harboured by *M. namaquensis* with a total of six species and one family being collected (Table 4). Unidentified trombiculid (chigger) mites were the most prevalent and abundant mite species followed by *A. rhabdomysi.* Unlike the fleas and lice, trombiculid mites occurred at high abundance. A total of 46 specimens of an unknown *Laelaps* sp. were found, consisting of five nymphs and 41 adults. The remaining two *Laelaps* spp. occurred at a substantially lower prevalence and abundance (Table 4).

Ticks comprised the greatest species diversity of the ectoparasite taxa found on *M. namaquensis*. They were represented by at least 8 species from three genera (Table 4). The larvae and nymphs of *Rhipicephalus warburtoni* and *Rhipicephalus arnoldi* resemble each other closely hence we chose to refer to them as *Rhipicephalus warburtoni*/*arnoldi*. Similarly, the immature stages of the *Haemaphysalis* spp. collected all belonged to the *Haemaphysalis* (*Rhipistoma*) group of species and have few distinguishing marks. Consequently, data for these species were pooled. Two nymphs that were allowed to moult were identified as *H. spinulosa*-like (Table 4). The immature stages of the genus *Ixodes* in Africa pose similar challenges as those outlined for the above species and were hence only identified to genus level. Only two of the tick species recovered occurred at significant numbers with *Rhipicephalus distinctus* being the most prevalent and abundant followed by *Haemaphysalis* (*Rhipistoma*) spp*.* (Table 4). The remaining tick species occurred at substantially lower prevalence and abundance.

### Effects of season and sex on ectoparasite distribution

#### Fleas

**Figure 1:**
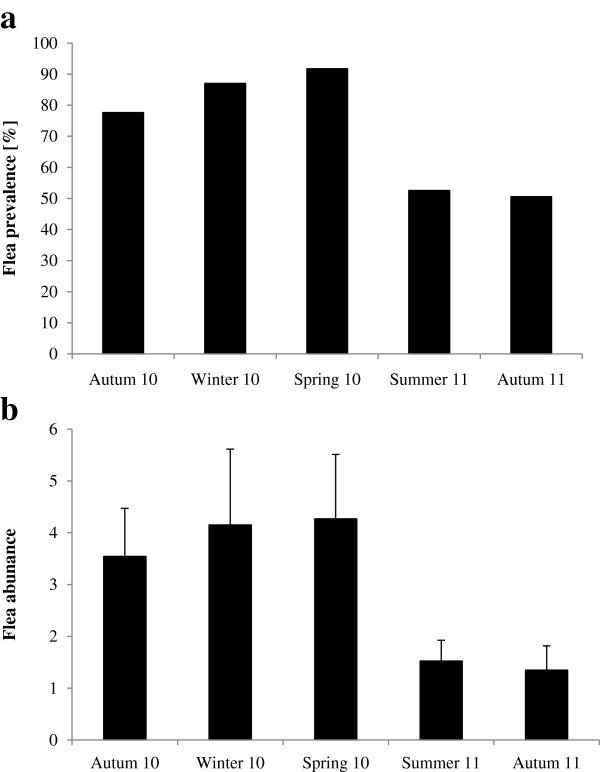
**Seasonal variation in flea a) prevalence and b) abundance of** ***M. namaquensis.***

Both total flea prevalence and abundance varied significantly with season (Table 5). Post-hoc analyses revealed that flea prevalence was significantly lower in summer compared to winter spring and autumn 2011 (p ≤ 0.015 for all comparisons). In addition, it was significantly lower in autumn 2011 compared to winter and spring (p ≤ 0.045 for both comparisons, Figure 1a). None of the remaining comparisons was significant (p ≥ 0.053 for all comparisons). In addition, none of the other factors considered was significant (Table 5).

**Table 5:** **Results of the GLMMs for total ectoparasite prevalence and abundance of Namaqua rock mice**

Taxon	Factors	df	Prevalence		Abundance	
			Waldχ ^***2***^	p	Waldχ ^***2***^	p
Fleas	Habitat	1,196	2.949	0.088	4.648	0.032*
	Season	4,196	3.874	0.005*	7.033	<0.0001*
	Sex	1,196	0.319	0.573	0.651	0.421
	Season × sex	4,196	0.263	0.901	0.333	0.855
	Body length	1,196	1.071	0.302	6.835	0.010*
Lice	Habitat	1,196	0.288	0.592	0.358	0.550
	Season	4,196	0.224	0.925	0.369	0.830
	Sex	1,196	0.000	0.997	0.000	1.000
	Season × sex	4,196	0.718	0.580	1.302	0.271
	Body length	1,196	2.856	0.093	3.338	0.069
Mites	Habitat	1,196	1.179	0.279	2.763	0.098
	Season	4,196	6.623	<0.0001*	37.991	<0.0001*
	Sex	1,196	0.305	0.581	0.033	0.857
	Season × sex	4,196	1.174	0.324	0.428	0.788
	Body length	1,196	0.014	0.907	1.336	0.249
Ticks	Habitat	1,196	0.101	0.751	2.399	0.123
	Season	4,196	4.036	0.004*	28.727	<0.0001*
	Sex	1,196	1.293	0.257	3.387	0.067
	Season × sex	4,196	0.178	0.949	2.096	0.083
	Body length	1,196	0.481	0.489	1.601	0.207

*Indicates significant results.

The flea abundance was significantly greater in autumn 2010 compared to summer and autumn 2011 (p ≤ 0.010 for both comparisons, Figure 1b). Similarly, it was significantly higher in spring than in summer and autumn 2011 (p ≤ 0.012 for both comparisons). In contrast, none of the remaining pairwise comparisons was significant (p ≥ 0.060 for all comparisons). The abundance of fleas was significantly lower in grassland (1.61 ± 0.65) compared to rocky outcrops (4.34 ± 0.78, Table 5). Neither host sex, nor the interaction between season and sex had a significant effect on flea abundance (Table 5). In contrast, the abundance of fleas increased with body length of the host (estimate: 0.025 ± 0.010).

#### Lice

The louse prevalence did not vary significantly with any of the factors considered (Table 5). Similarly, none of the independent variables significantly affected louse abundance (Table 5).

#### Mites

**Figure 2:**
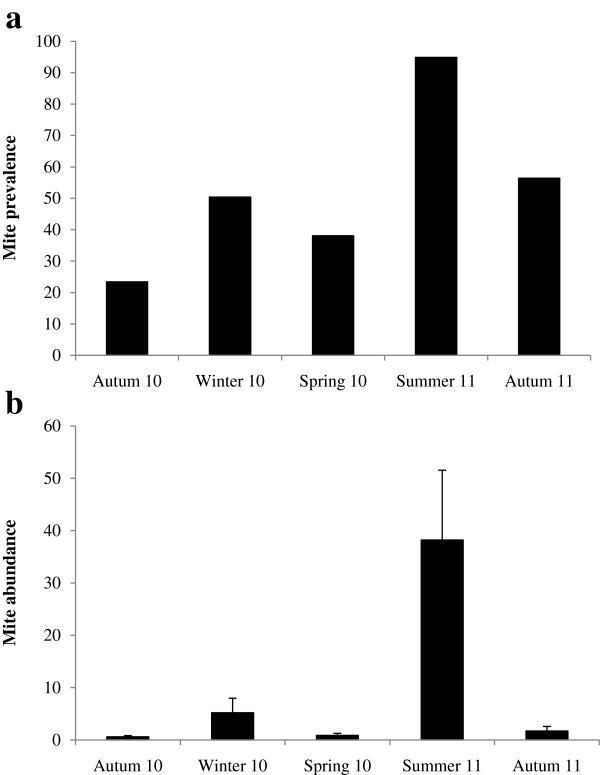
**Seasonal variation of mite a) prevalence and b) abundance of** ***M. namaquensis*** **.**

Both total mite prevalence and abundance varied significantly with season (Table 5). Mite prevalence was greatest in summer and it was significantly higher compared to all other seasons (p ≤ 0.034 for all comparisons, Figure 2a). In addition, it was significantly lower in autumn 2010 compared to autumn 2011 (t = 2.124, p = 0.034). None of the remaining pairwise comparisons was significant (p ≥ 0.100 for all comparisons). Neither sex, habitat type, body length nor the interaction between sex and season had a significant effect on mite prevalence (Table 5).

Mite abundance was significantly higher in summer compared to all other seasons (p ≤ 0.013 for all comparisons, Figure 2b). No other pairwise comparisons were significant (p ≥ 0.117 for all comparisons). In addition, none of the other factors considered was significant (Table 5).

#### Ticks

**Figure 3:**
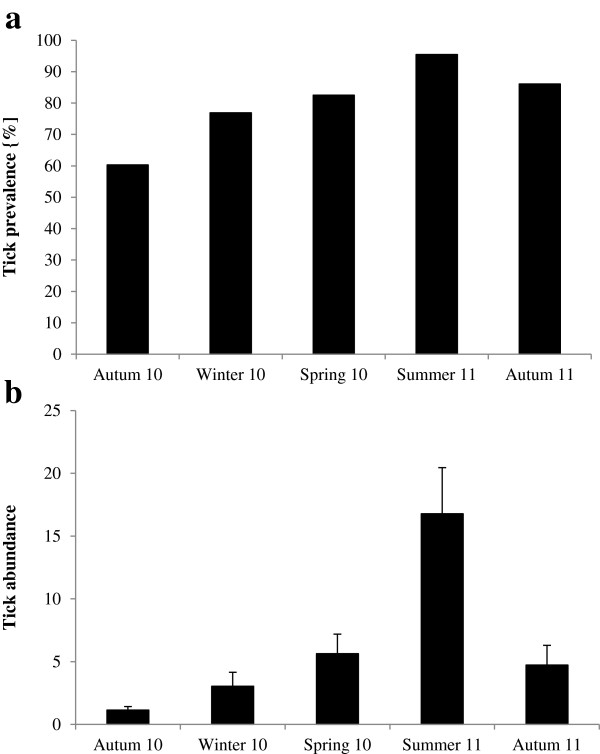
**Seasonal variation of tick a) prevalence and b) abundance of** ***M. namaquensis*** **.**

Both the tick prevalence and the abundance varied significantly with season (Table 5). The prevalence was significantly lower in autumn 2010 than in spring, summer and autumn 2011 (p ≤ 0.015 for all comparisons, Figure 3a). None of the remaining pairwise comparisons was significant (p ≥ 0.125 for all comparisons). In addition, none of the other factors considered significantly affected the tick prevalence (Table 5).

The abundance of ticks was lowest in autumn 2010 and highest in January 2011 (Figure 3b). Accordingly, it was significantly lower during autumn 2010 compared to spring, summer and autumn 2011 (p ≤ 0.007 for all comparisons). In contrast, tick abundance in summer was significantly greater than during all other seasons (p ≤ 0.001 for all comparisons). None of the remaining pairwise comparisons between seasons was significant (p ≥ 0.095 for all comparisons). Furthermore, none of the other factors considered had a significant effect on tick abundance (Table 5).

## Discussion

In the current study individuals from eleven small species were collected. However, *M. namaquensis* far outnumbered the other species, irrespective of whether total counts or habitat-specific results are considered. This finding corroborates previous reports that the study species has a wide habitat tolerance [32] and hence may also be common in the vicinity of human settlements where they might be hosts for vectors of medical and veterinary concern. Nevertheless, capture numbers for *M. namaquensis* were more than fourfold higher on rocky outcrops suggesting that this habitat type is particularly suitable for the study species. However, the difference in animal numbers was not limited to the study species and capture numbers for all small mammals species combined were more than four times as high on rocky outcrops compared to grassland. At the same time the species composition for the two habitat types differed substantially with four species being unique to grassland (*T. leucogaster*, *R. dilectus*, *Dendromys* spp. and *M. minutoides*), while another four were exclusively caught on rocky outcrops (*Acomys spinosissimus*, *Aethomys ineptus*, *Graphiurus* spp.) suggesting that these species have narrower habitat tolerances.

We found a great diversity of ectoparasites harboured by *M. namaquensis* with 23 species from four taxa, of which ticks were the most speciose. Compared to the total species diversity of no less than 74 ectoparasite species recorded for *M. namaquensis* this may appear impoverished. However, the current study constitutes the first systematic long-term assessment of the ectoparasite community of *M. namaquensis* from a single locality and hence this ectoparasite diversity is rather remarkable. Furthermore, our data confirm that with regards to prevalence the two taxa with the highest vector potential, namely fleas and ticks, are the most important ectoparasite taxa sustained by *M. namaquensis*.

All fleas collected in the current study are widely distributed throughout southern Africa [37], however, only *X. brasiliensis* is of medical significance. It is characterised by a low host specificity and thought to be a plague vector and can carry *Bartonella* spp. [9, 12, 37, 49]. In contrast, *C. godfreyi* and *E. aganippes* appear to be specific for *M. namaquensis* [37] and hence the transmission potential of zoonotic diseases to other species is likely to be low. Nonetheless, apart from zoonotic *Bartonella* spp. closely related to *B. elizabethae*, host-specific *Bartonella* spp. have been found in *M. namaquensis* [44, 45] and it is possible that these are transmitted by such host-specific vectors. *Demeillonia granti* is host-specific for sengis (Macroscelididae), while the principal hosts of *D. ellobius* are grassland rodents such as gerbils (*Tatera* spp.), the multimammate mouse (*Praomys natalensis*) and the four-striped mouse (*Rhabdomys pumilio*) and hence the observed infestation is probably accidental [37, 50, 51]. This is corroborated by the low prevalence and abundance of these species on *M. namaquensis*.

Little is known about the lice collected in this study but all have previously been found on the study species or the closely related red veld rat (*Aethomys chrysophilus*) [31, 49]. Given the recent revision of the genus and the overlap in the distributional range this may also have been the Tete veld rat (*A. ineptus*) that was also caught during the current study. The restricted host range reported for these lice species so far may indicate that they are specific for the genera *Aethomys* and *Micaelamys*.

Chiggers are known for their low host specificity and were encountered at high abundances in the current study. Various chigger species can cause skin irritations and have been implicated as vectors for *Rickettsia* and *Toxoplasma* in the Ethiopian region [31, 52]. However, nothing is known about the validity of this assumption for the study region [31]. The other common mite species (*A. rhabdomysi*) has only recently been described from *R. pumilio* in the Western Cape [53] and hence the current finding constitutes a new host as well as locality record. It was substantially more prevalent and abundant on *M. namaquensis* compared to its nominal host suggesting that *M. namaquensis* may be a more important host for this mite than *R. pumilio*. The remaining mite species have been reported for a number of rodent species and appear to show little host specificity with the possible exception of *L. simillimus* [39, 54]. For *L. roubaudi* this is a new host record and it has previously only been reported from the DRC, Nigeria and Ghana [54].

We collected several *Rhipicephalus* spp. of veterinary and/or medical importance including *R. warburtoni*, *R. evertsi evertsi*, *R. decoloratus* as well as two immatures of the economically most important *Rhipicephalus* spp. in Africa *R. appendiculatus* [13, 30, 55–57]. However, like *Ixodes* spp. all of these occurred at low prevalence. With the exception of *R. warburtoni* and *R. arnoldi* this is possibly linked to the preference of most of these ticks for hosts living in grassland habitats rather than the rocky outcrops preferred by *M. namaquensis*. Consequently, most of these infestations can be considered accidental and *M. namaquensis* is unlikely to play a major role in maintaining these ticks or act as an important reservoir for any of the diseases they transmit. In fact, it has been shown that *M. namaquensis* is a poor host for *Ixodes rubicundus* (causing sheep paralysis) and *R. warburtoni* (carrying *A. bovis* and causing goat paralysis) and these tick species exhibit a preference for sympatrically occurring eastern rock sengis (*Elephantulus myurus*) as hosts [28–30, 58, 59]. In contrast, *R. distinctus* was the main tick species found on *M. namaquensis* at a high prevalence and abundance. As with *R. arnoldi*, little is known about this tick and substantial numbers have previously been reported from rock hyraxes (Hyracoidea) and rock rabbits (*Pronolagus* spp.) suggesting that these ticks share the habitat preferences of the *M. namaquensis* [13]. The vector status of both of these species is unknown but deserves attention in the future. Conversely, *Haemaphysalis* (*Rhipistoma*) *elliptica*, a member of the second most prevalent and abundant tick *Haemaphysalis* (*Rhipistoma*) spp. group, is of great veterinary and medical importance as it transmits *B. rossi*, the cause of the most important and virulent tick-borne disease of domestic dogs in South Africa [42, 60] and carries *R. conorii* causing African tick bite fever. Namaqua rock mice appear to be a preferred host for *H. elliptica* [28–30] and consequently the large prevalence of *Haemaphysalis* (*Rhipistoma*) spp. recorded in the current study suggests that *M. namaquensis* may play an important role in the disease dynamic, a hypothesis that should be addressed in future studies.

With the exception of fleas, habitat type did not affect ectoparasite burdens observed in the study species. This may be linked to differences in *M. namaquensis* density, small mammal species composition between the two habitats and parasite taxon life-cycles that can result in similar outcomes. Since fleas are mostly directly transmitted, the lower number of host individuals in grassland compared to rocky outcrops may reduce transmission rates in the former habitat. However, studies in other African small mammal species did not find an effect of host population density within the same habitat type on flea burden [61, 62]. These were, however, based on comparisons within the same habitat type and small mammal species assemblages did not differ between densities in these studies. In contrast, species composition differed substantially between the two habitat types in our study and if any of the grassland specialist species is either a preferred host for fleas or acts as an ecological trap removing fleas from the system [63] this could account for the observed differences. Although large numbers of fleas have been reported to infest *T. leucogaster* and *Rhabdomys* spp. [27, 49] this hypothesis is currently speculative and deserves further attention in the future. Unlike fleas, ticks and the most common mites (chiggers) are not transmitted between hosts. The lack of habitat effects for these two taxa could be caused either by host behaviour and/or their abundance in the environment. If parasite density in the habitat differs between habitat types larger home ranges in grasslands with lower *M. namaquensis* numbers would result in comparable exposure rates in both habitats. Similarly, greater densities of these two taxa on rocky outcrops would lead to similar exposure rates in both habitats. Although we cannot distinguish these possibilities from the current data, support for the latter hypothesis comes from other studies which show that tick burden is a function of tick abundance rather than host density [64, 65]. Despite their direct mode of transmission, the low mobility and abundance may account for habitat effects on louse burdens.

Seasonal patterns in prevalence and abundance were apparent for all ectoparasite taxa collected in this study with the exception of lice. This might be related to differences in the life-cycle of the different taxa. Unlike fleas, mites and ticks, lice usually spend their entire life on the host and are thus likely to be less affected by climatic factors than the other taxa which spend substantial amounts of their life-cycle off-host [16–18, 20]. Such differences in seasonal patterns of various ectoparasite taxa have previously been observed in other rodent hosts in South Africa [27, 49, 66]. For fleas, mites and ticks seasonal peaks in abundance and prevalence coincided with the wet period of the year from October to April when rainfall may greatly reduce the risk of desiccation for these ectoparasites when they are not feeding on the host [19]. However, this peak occurred earlier for fleas (i.e. spring) than for mites and ticks (summer). Given that most fleas and mites spend substantial amounts of their lives in the nests of their hosts while ticks usually quest in the environment, the difference between fleas and mites is unexpected. However, the dramatic increase in mite abundance observed in summer was largely attributable to an increase in the abundance of chiggers (D.M. Fagir, personal observation) that are soil dwelling [52]. Consequently, the susceptibility of these mites may be expected to be more similar to that of the tick species in this study than to other mite species or fleas and chiggers may only hatch in response to rainfall that started in spring. In addition, as ectoparasites dwelling in the host’s nest fleas may be able to respond immediately to changes in immunity in their host as a result of reproductive activity and the onset of breeding in the study species that commences in September [35].

None of the parasite taxa found in the current study exhibited a sex-bias. If the distribution of ectoparasites in *M. namaquensis* is body size dependent the absence of a sexual dimorphism in the study species [33] this study] may account for this observation. The lack of body length effects for three of the four taxa provides corroborating evidence for this hypothesis. In addition, female *M. namaquensis* appear to be more sedentary and use smaller home ranges than males [34], suggesting that body size rather than behavioural differences accounts for this observation. Also, it has been stressed recently that though frequently assumed sex-biases in ectoparasite burden are not as common as was previously thought [67].

The smaller size of younger animals may account for the observation that flea abundance increased with body size in the study species. Similar correlations between body size and flea burden have been reported for other rodent species and have proposed that resource size (i.e. host size) may be the determining factor for this relationship [68, 69]. However, this does not appear to be the case for other parasite taxa and [68, 69] we did not find evidence for a similar relationship in ticks and mites and their abundance appears to be rather a function of parasite abundance in the environment than host factors [64, 65]. The latter hypothesis needs to be addressed in the future.

## Conclusion

*M. namaquensis* were the dominant small mammal species in the study area irrespective of habitat type. They harboured 23 ectoparasite species from four taxa in the study area with fleas and ticks being the most important ones with regards to prevalence and abundance. Although many of these are known vectors for diseases of veterinary and/or medical importance *M. namaquensis* is likely to play a significant role as vector host for one flea (*X. brasiliensis*) and one tick species (*Haemaphysalis* spp.) only. All ectoparasite taxa exhibited seasonal peaks in abundance coinciding with the warm and wet period of the year while no effects of host sex were observed and body size effects were only apparent for fleas. Additional research addressing the role of the study species as reservoir for zoonotic diseases in southern Africa is urgently needed.
